# spread.gl: visualizing pathogen dispersal in a high-performance browser application

**DOI:** 10.1093/bioinformatics/btae721

**Published:** 2024-11-29

**Authors:** Yimin Li, Nena Bollen, Samuel L Hong, Marius Brusselmans, Fabiana Gambaro, Joon Klaps, Marc A Suchard, Andrew Rambaut, Philippe Lemey, Simon Dellicour, Guy Baele

**Affiliations:** Department of Microbiology, Immunology and Transplantation, Rega Institute, KU Leuven, Leuven 3000, Belgium; Spatial Epidemiology Lab (SpELL), Université Libre de Bruxelles, Brussels 1050, Belgium; Department of Microbiology, Immunology and Transplantation, Rega Institute, KU Leuven, Leuven 3000, Belgium; Spatial Epidemiology Lab (SpELL), Université Libre de Bruxelles, Brussels 1050, Belgium; Department of Microbiology, Immunology and Transplantation, Rega Institute, KU Leuven, Leuven 3000, Belgium; Department of Microbiology, Immunology and Transplantation, Rega Institute, KU Leuven, Leuven 3000, Belgium; Spatial Epidemiology Lab (SpELL), Université Libre de Bruxelles, Brussels 1050, Belgium; Department of Microbiology, Immunology and Transplantation, Rega Institute, KU Leuven, Leuven 3000, Belgium; Department of Biomathematics, David Geffen School of Medicine, University of California Los Angeles, Los Angeles, CA 90095, United States; Department of Biostatistics, Fielding School of Public Health, University of California Los Angeles, Los Angeles, CA 90095, United States; Department of Human Genetics, David Geffen School of Medicine, University of California Los Angeles, Los Angeles, CA 90095, United States; Institute of Ecology and Evolution, University of Edinburgh, Edinburgh, EH9 3FL, United Kingdom; Department of Microbiology, Immunology and Transplantation, Rega Institute, KU Leuven, Leuven 3000, Belgium; Department of Microbiology, Immunology and Transplantation, Rega Institute, KU Leuven, Leuven 3000, Belgium; Spatial Epidemiology Lab (SpELL), Université Libre de Bruxelles, Brussels 1050, Belgium; Department of Microbiology, Immunology and Transplantation, Rega Institute, KU Leuven, Leuven 3000, Belgium

## Abstract

**Motivation:**

Bayesian phylogeographic analyses are pivotal in reconstructing the spatio-temporal dispersal histories of pathogens. However, interpreting the complex outcomes of phylogeographic reconstructions requires sophisticated visualization tools.

**Results:**

To meet this challenge, we developed spread.gl, an open-source, feature-rich browser application offering a smooth and intuitive visualization tool for both discrete and continuous phylogeographic inferences, including the animation of pathogen geographic dispersal through time. Spread.gl can render and combine the visualization of multiple layers that contain information extracted from the input phylogeny and diverse environmental data layers, enabling researchers to explore which environmental factors may have impacted pathogen dispersal patterns before conducting formal testing. We showcase the visualization features of spread.gl with representative examples, including the smooth animation of a phylogeographic reconstruction based on >17 000 SARS-CoV-2 genomic sequences.

**Availability and implementation:**

Source code, installation instructions, example input data, and outputs of spread.gl are accessible at https://github.com/GuyBaele/SpreadGL.

## 1 Introduction

Statistical phylogeographic inference has become widely adopted as a method to investigate viral circulation, often offering the ability to reconstruct the migration history of pathogens in a spatial-temporal context. Phylogeographic models and methods have been implemented in various software packages, each using its own inference procedures. BEAST 1.10 (Suchard *et al.* 2018) and BEAST 2 (Bouckaert *et al.* 2019) are widely used software packages for Bayesian phylogeographic analysis, whereas TreeTime (Sagulenko *et al.* 2018) and PastML (Ishikawa *et al.* 2019) use maximum-likelihood inference to infer ancestral discrete locations. While the use of discrete phylogeographic inference may be more widespread than its continuous counterpart, the applicability and performance of both approaches remain a topic of continued investigation (Kalkauskas *et al.* 2021, Layan *et al.* 2023), which will undoubtedly lead to further improvements and novel implementations.

To visualize and interpret the outcomes of phylogeographic analyses, a range of software packages have been developed to parse location-annotated phylogenies, which contain spatial and temporal information, making them well-suited to create animations through time. Arguably, the most popular (online) visualization software package resource in recent years is Nextstrain (Hadfield *et al.* 2018). Auspice is an open-source interactive tool that powers the visualizations within Nextstrain, allowing customization of aesthetics and functionality, making use of associated metadata such as geographic information, serology, or host species. Nextstrain offers a visual interface that consists of three linked panels: a phylogenetic tree, the inferred transition events among locations, and the genetic diversity across the pathogen’s genome. PastML (Ishikawa *et al.* 2019) enables a visualization through a zoomable HTML map while performing a two-step compression: a vertical compression clusters together the regions of the tree where no state changes occur, whereas a horizontal compression merges identical subtree configurations. SPREAD (Bielejec *et al.* 2011), SpreaD3 (Bielejec *et al.* 2016), and SPREAD4 (Nahata *et al.* 2022) are a set of successive improvements of software used to analyse and visualize the results of phylogeographic reconstructions. A short overview of other visualization applications is discussed in the recent SPREAD4 publication (Nahata *et al.* 2022). Certain visualization approaches, such as the one implemented in the R package ‘seraphim’ (Dellicour *et al.* 2016) or the Python library ‘baltic’ (https://github.com/evogytis/baltic), offer great flexibility but require programming knowledge to visualize a pathogen’s dispersal history.

Each of the tools mentioned above has its own advantages and disadvantages, the latter of which may be inflexible customization, unintuitive user interfaces, as well as compatibility and dependency issues. For example, SPREAD relied on the Google Earth software and as such required converting a consensus tree or posterior set of trees to Keyhole Markup Language (KML). Its successor, SpreaD3, no longer relied on external software nor on having an internet connection but became sensitive to updates to many of its supported browser platforms. SPREAD4, a cloud-based successor of SpreaD3, currently uses Amazon Web Services for its calculations and features account-based storage and easy sharing of visualizations by means of unique web addresses. However, it is still inconvenient for users to adjust elements of its phylogeographic animations, such as the start and end times, the current time point, and the playback speed. Furthermore, a number of visualization packages focus on a specific type of phylogenetic inference (i.e. discrete or continuous), and may hence not offer support for both types. Apart from these issues, most applications struggle to visualize phylogeographic reconstructions of large data, an issue that became all too apparent when studying the spread of SARS-CoV-2 lineages. When a large phylogenetic tree is loaded, a range of potential issues can emerge, such as long processing times, unsatisfactory responsiveness and obvious animation stuttering.

To address the various limitations mentioned above, we have developed spread.gl, a high-performance open-source browser-based visualization application. This latest instalment in the continuing SPREAD development focuses on smooth and high-performance animations, improved aesthetics and extensive functionality. A key focus of this new version is to enable the simultaneous visualization of many data layers, with a typical use case being the inclusion of environmental data layers upon which a pathogen’s dispersal through time and space can be visualized. Further, the order of these different types of layers is made adjustable and can be easily enabled or disabled. We use these various (types of) data layers to incorporate environmental and ecological factors that offer potentially important context for the dispersal history and spatio-temporal patterns of pathogens, which is not possible in the other software packages mentioned above. We showcase the capabilities of spread.gl through pathogen dispersal examples in the *Visualization examples* section.

## 2 Software features

To accomplish the aforementioned functionalities, spread.gl was built upon kepler.gl—a powerful open-source framework for geospatial analysis of big data—to enable the efficient rendering of large-scale phylogenies on maps with highly customizable visualization options. kepler.gl—and by extension spread.gl—is a client-side-only application, meaning that the data you upload to the application are only stored in the browser and no information or map data is sent to any server, which is of particular interest when working with health data or any type of data with privacy concerns. Three different data formats are accepted as input: comma-separated values (CSV), JavaScript object notation (JSON), and GeoJSON, an open standard format designed for representing geographical features, along with their nonspatial attributes. Phylogenetic trees output from Bayesian phylogeographic analyses are typically stored as NEXUS files so we provide a way to convert them into an acceptable input data format. A key step consists of creating an *arc layer* to display the phylogenetic branches, combined with additional layers to visualize important elements related to the ancestral location nodes from discrete or continuous phylogeographic inference.

An important addition in spread.gl, compared to previous versions of SPREAD, is the ability to visualize phylogeographic reconstructions on top of an environmental data layer to enable a visual exploration of possible associations with pathogen dispersal. Construction of an environmental layer is relatively straightforward (see the online tutorial mentioned in the ‘Data availability’ section), and several such layers can be combined within a single visualization. Once all the layers of interest have been prepared and added, an animation of pathogen dispersal can be generated and played over an incremental or moving time window.

When a visualization has been constructed to the user’s satisfaction, the final result can be exported as an image or a JSON file, an HTML file—which can easily be shared with others who are not required to have spread.gl installed—or captured from the screen using a video capture tool. The final result of spread.gl can also be exported as a PDF using a browser extension such as GoFullPage (Coles 2012), which is free to use and compatible with different browsers, e.g. Google Chrome and Microsoft Edge. Safari on MacOS allows to directly save the web page as a PDF.

In the examples below, we focus on parsing the output from the BEAST 1.10 (Suchard *et al.* 2018) and BEAST 2 (Bouckaert *et al.* 2019) software packages, arguably the most widely used tools for studying a pathogen’s spatio-temporal dispersal patterns. The output of other software packages can of course also be used, as long as the output format is the same, or is able to be converted using one of the corresponding processing scripts (see the ‘Data availability’ section).

### 2.1 Visualization of discrete phylogeographic inference

When visualizing the outcome of a discrete phylogeographic analysis, in addition to displaying the phylogenetic branches using an *arc layer*, one can create an additional *cluster layer* to emphasize the relevance of each ancestral location in the pathogen’s dispersal history. Such a layer attaches a circle centred on each ancestral location, with a radius proportional to the number of lineages present in that location. This cluster layer not only visually represents the varying intensity at each location of pathogen spread based on the accumulated ancestral lineages in a specific period, but also provides additional details of absolute counts by hovering the mouse over the circles. We also provide a Bayes factor test to identify well-supported rates between locations by estimating their statistical support through Bayesian stochastic search variable selection (BSSVS) (Lemey *et al.* 2009). Once the Bayes factors have been computed (see the online tutorial link in ‘Data availability’ section), they can be added as a filter in spread.gl so as to only display the transition rates that are well-supported by the data.

### 2.2 Visualization of continuous phylogeographic inference

When visualizing the outcome of continuous phylogeographic inference, the *arc layer* that represents the phylogenetic branches can be combined with a *point layer* to show the sampled genomes as well as their ancestral locations. Furthermore, a *contour layer* comprised of semi-transparent polygons can be created to depict the highest posterior density (HPD) region for each ancestral node location and to visually represent the geographic uncertainty of the location estimates (Lemey *et al.* 2010).

### 2.3 Visualization of large-scale phylogeographic inference

Large-scale phylogenetic and phylogeographic analyses present a myriad of potential processing problems, including how to perform (any type of) Bayesian phylogeographic inference and how to generate maximum clade credibility (MCC) trees from the large output files that contain annotations for thousands or tens of thousands of ancestral nodes. As such, the output phylogeny from such large-scale studies may—by necessity—be a single (e.g. the final) location-annotated phylogeny sampled from the posterior distribution [see e.g. Kraemer *et al.* (2021)] without any uncertainty estimates for the estimated locations at the internal nodes. During the visualization process, such a single tree may not be easily processed as it typically stores the exact geographical coordinates of each location within a single annotation, rather than as two distinct continuous traits in a standard MCC tree. Additionally, the use of various coordinate reference systems (CRS) necessitates specific programming expertise in geographic coordinate conversion, which can be challenging for users to implement proper re-projection on the map. spread.gl is designed to address the challenges described in these scenarios, enabling the smooth and responsive visualization and animation of large data.

### 2.4 Visualization of environmental layers

When studying the spread and evolution of pathogens, it is often of interest to investigate which environmental factors may potentially influence their dispersal. To facilitate such a visual exploration in spread.gl, we provide the opportunity to overlay environmental and/or ecological layers. Such data are often available in the public domain and can be freely downloaded from online resources. There are two primary types of these data, i.e. raster and vector data, both of which are supported in the spread.gl toolkit. Raster data come in the form of a geo-referenced matrix where each cell represents a geographic region and contains an attribute value that measures a particular characteristic of that region. This measurement can be discrete, to represent distinct categories such as land cover variables, or continuous, to store gradually changing values such as temperature and elevation. Vector data consist of different geometric features and multiple assigned attributes that store additional information, with GeoJSON being one of the most general vector data formats, which is supported in spread.gl. To assign such data to the locations of interest, the actual shapes of the locations of interest (e.g. countries, provinces, etc.) need to be retrieved. Shape data are also typically freely available online either in a shape file or in the GeoJSON format. Both a *point layer* and a *polygon layer* can be used in spread.gl to visualize these additional layers. For example, a *point layer—*with a customizable radius of the points—can be used to show gradually changing environmental data, whereas a *polygon layer* is typically used to show values—e.g. the gross domestic product (GDP)—for larger administrative areas.

## 3 Visualization examples

In the sections below, we introduce three examples of phylogeographic studies and showcase the visualization of the inference results in spread.gl. These constitute the primary use cases for spread.gl but there are many more custom visualizations that can be created (see the GitHub repository and the provided example HTML output files as mentioned in the ‘Data availability’ section).

### 3.1 SARS-CoV-2 lineage B.1.1.7 in England (large-scale continuous phylogeography)

**Figure 1.**
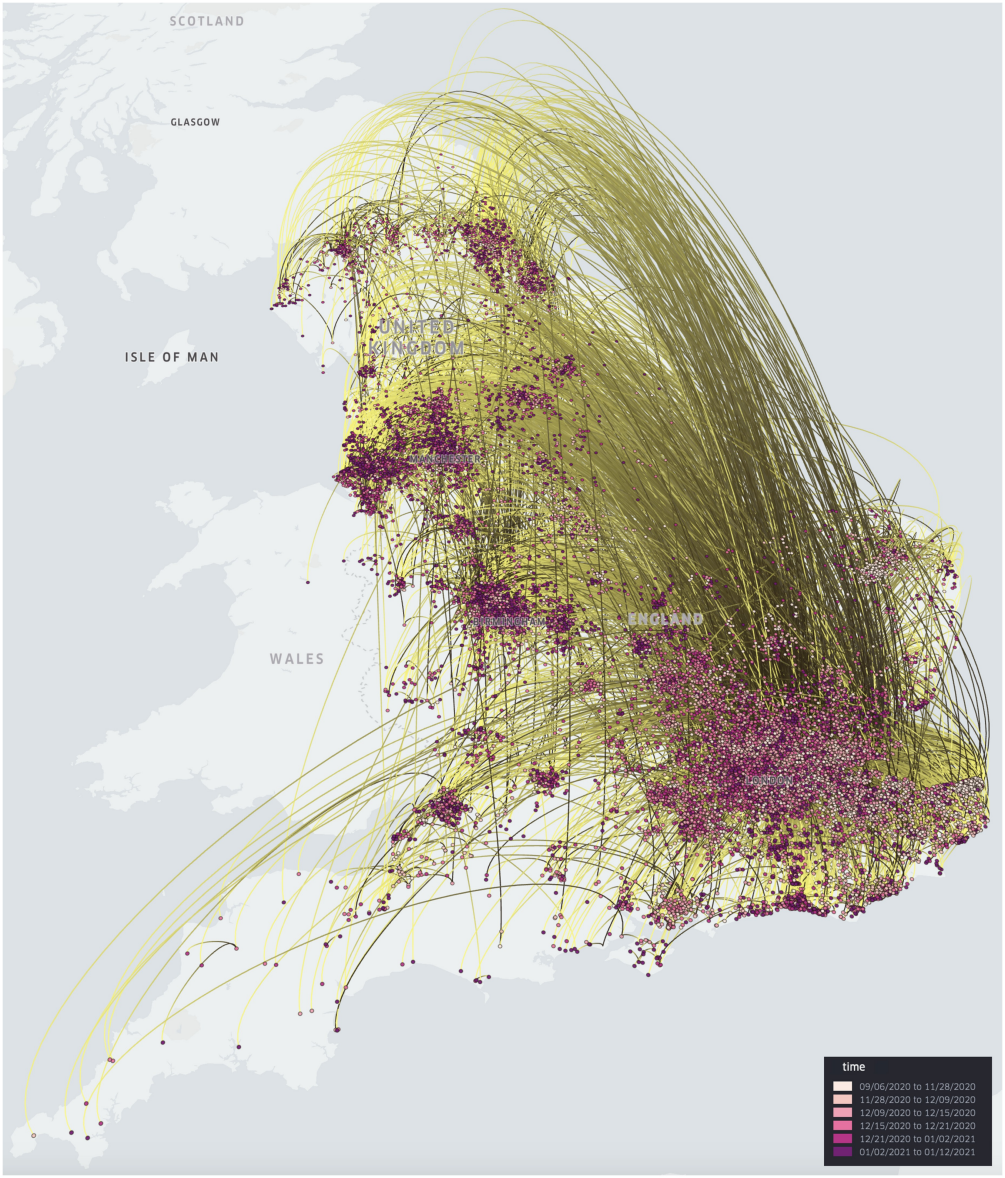
Continuous phylogeographic reconstruction of the dispersal history of SARS-CoV-2 lineage B.1.1.7 in England (in 3D mode), from 6 September 2020 until 12 January 2021. This visualization of a large SARS-CoV-2 dataset containing 17 203 genomes can be rendered efficiently using spread.gl, with animation of the spread over time being shown smoothly despite a large number of branches in the phylogeny.

In a study conducted by Kraemer *et al.* (2021), the dispersal history and dynamics of the B.1.1.7 lineage of severe acute respiratory syndrome coronavirus 2 (SARS-CoV-2) were analysed using a continuous phylogeography approach based on 17 716 genomes. This particular variant was initially identified in Kent or Greater London in September 2020 and rapidly spread throughout the United Kingdom by the end of that year, eventually becoming a variant of global concern. To visualize the continuous phylogeographic reconstruction from this study in spread.gl, we have performed the following steps. First, to achieve the correct projection, we re-projected the original coordinates by converting the CRS from the British National Grid (BNG) to the current World Geodetic System (WGS84) (to this end, a script is available in the GitHub repository of spread.gl). As a second step, we meticulously identified and removed outliers, which were data points that appeared outside England in the initial visualization, as these points lacked reliable geospatial information in the metadata. As a result, the total number of visualized genomes was reduced to 17 203. Finally, we created a *point layer* and an *arc layer* to represent the spatio-temporal information in the location-annotated phylogeny, for which we took the final sample from the original Bayesian continuous phylogeographic analysis. The end result is displayed in Fig. 1 (see Supplementary Figs S1–S4 for additional time points). By smoothly rendering an animated display of the many sampling points and phylogenetic branches, spread.gl showcases its high performance and robust support for handling large-scale datasets.

### 3.2 YFV in Brazil (continuous phylogeography with environmental data)

**Figure 2.**
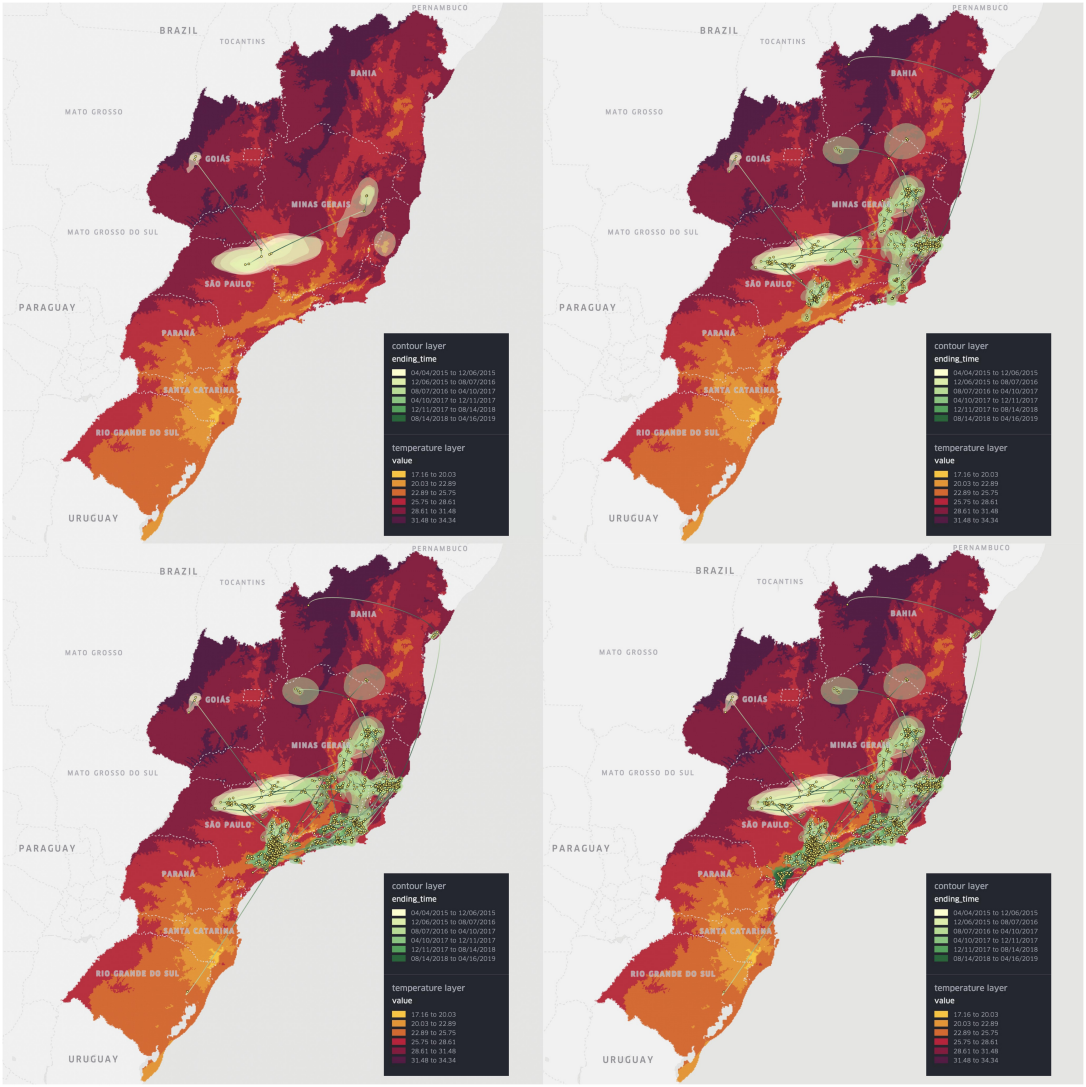
Continuous phylogeographic reconstruction of the dispersal history of yellow fever virus lineages in Brazil (in 2D mode) on top of a (maximal) temperature data layer: (a) until 7 April 2016; (b) until 10 April 2017; (c) until 12 April 2018; (d) until 16 April 2019. Specifically, we map the maximum clade credibility (MCC) tree and 80% highest posterior density (HPD) regions reflecting the uncertainty related to the Bayesian continuous phylogeographic inference. The MCC tree and 80% HPD regions are based on 1000 trees sampled from the posterior distribution of trees and are coloured according to their time of occurrence using a yellow-green colour scale.

Hill *et al.* (2022) conducted an analysis of the outbreak of yellow fever virus (YFV) in Brazil between 2016 and 2019. The authors reconstructed the spatio-temporal spread based on 705 YFV sequences, generated from neotropical primate, human and mosquito samples. This study considered the effects of environmental factors on the spread of YFV and identified a significant association between temperature and virus effective population sizes. We downloaded historical maximum temperature data from WorldClim (Fick and Hijmans 2017, Harris *et al.* 2020) and created a *point layer* of the average maximum temperatures across the area that covers the relevant Brazilian states during the virus outbreak. To balance file size and granularity, temperature data with a spatial resolution of 2.5 min (∼21 km^2^ at the equator) were used in our visualization. The resulting raster file was clipped using a mask layer consisting of a boundary map and a list of locations, to display only the Brazilian states of interest in the spread.gl visualization. On top of this environmental layer, we created a *point layer*, an *arc layer*, and a *contour layer* to represent the spatio-temporal information (and its associated uncertainty) in the location-annotated MCC tree (Lemey *et al.* 2010). In Fig. 2, we show the complete spread.gl visualization of the continuous phylogeographic reconstruction on top of an environmental data layer representing the global maximum temperature averaged over all months during the outbreak, which corresponds to an environmental factor suspected to impact the genetic diversity and dispersal dynamics of the virus (Hill *et al.* 2022). Improving the transparency of the contour layer (shown as transparent yellow-green polygons), achieved by reducing the opacity of the polygons from a standard 20% to a <5%, ensures that the temperature layer is not excessively obscured to facilitate a clearer understanding of the dynamics between temperature and state transitions.

### 3.3 PEDV in China (discrete phylogeography with environmental data)

**Figure 3.**
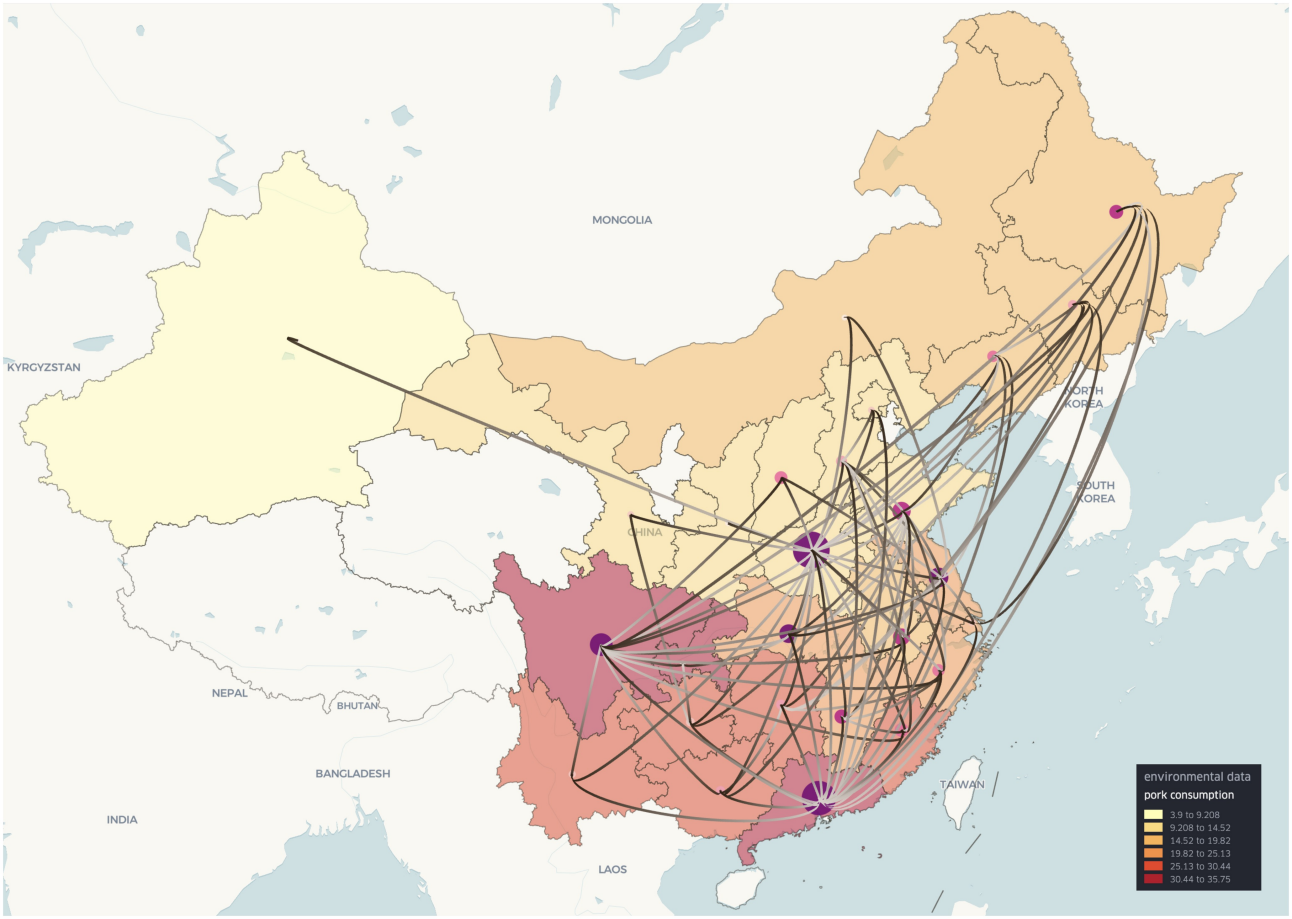
Discrete phylogeographic reconstruction of the spread of porcine epidemic diarrhoea virus in China (in 2D mode) on top of a semi-transparent data layer that shows pork consumption per province. Provinces for which these data are not available are not visualized. Colours towards the yellow spectrum indicate lower pork consumption, whereas colours towards the (dark) red spectrum indicate higher pork consumption. The start and end of the branches are coloured in white and grey, respectively, with the colour gradually changing across the length of the branches. Darker purple clusters represent locally higher cumulative lineage counts over a custom period, while lighter pink clusters represent lower cumulative counts.

Porcine epidemic diarrhoea virus (PEDV) generally results in high mortality in infected suckling pigs. To study the dispersal pattern of this virus, He *et al.* (2022) conducted a discrete phylogeographic analysis of a dataset that contained 769 sequences sampled in 26 provinces across China and unravelled the spread of PEDV genotype G2 during the past two decades. In their study, the authors explored a range of environmental factors of interest to determine which of them had an important impact on the dispersal of PEDV between provinces in China. We first curated an environmental layer as a *Geojson layer* in spread.gl, containing 16 environmental variables of interest related to economy, demography and geography for each province: sample size, slaughtered pigs, pork price, live pig price, pork consumption, total consumption, feed price, feed production, GDP, human population size, human population density, maximum elevation, mean elevation, mean temperature, mean vapour pressure, and mean precipitation. As shown in Fig. 3, each province can be coloured based on the corresponding value of an environmental variable, in this case, mean pork consumption per capita (kg) between 2015 and 2018. On top of this environmental layer, we displayed an *arc layer* to show the phylogenetic branches and a *cluster layer* to reflect the relevance of each ancestral location in the pathogen’s dispersal. Based on an intuitive visual assessment, pork consumption does not show a pronounced impact on the spread of PEDV in China, although it was described as relevant to and indirectly associated with viral transmission in the original study (He *et al.* 2022). Indeed, from the environmental layer in Fig. 3, we observe that most of the provinces in South China have high pork consumption. However, from the reconstruction of PEDV viral spread, we observe that both Guangdong and Henan (i.e. not provinces with high pork consumption) are the main hubs for the spread of PEDV within China, followed by Sichuan (note that country, province and city names will appear automatically in spread.gl when zooming in on the map). These observations point to the importance of combining visual inspections with detailed statistical assessments to determine the impact of predictors on viral spread.

## Supplementary Material

btae721_Supplementary_Data

## Data Availability

All the required source code and installation instructions for spread.gl can be found on GitHub, as well as video animations of the visualizations presented in this article: https://github.com/GuyBaele/SpreadGL. HTML output from the spread.gl visualizations shown in this article can be readily downloaded from: https://github.com/GuyBaele/SpreadGL/tree/main/html. All the required input data and processing scripts—along with instructions on how to use them—for the spread.gl visualizations shown in this article can be found on: https://github.com/GuyBaele/SpreadGL/tree/main/inputdata.

## References

[btae721-B1] Bielejec F , Baele G, Vrancken B et al SpreaD3: interactive visualization of spatiotemporal history and trait evolutionary processes. Mol Biol Evol 2016;33:2167–9. 27189542 10.1093/molbev/msw082 PMC6398721

[btae721-B2] Bielejec F , Rambaut A, Suchard MA et al SPREAD: spatial phylogenetic reconstruction of evolutionary dynamics. Bioinformatics 2011;27:2910–2. 21911333 10.1093/bioinformatics/btr481 PMC3187652

[btae721-B3] Bouckaert R , Vaughan TG, Barido-Sottani J et al Beast 2.5: an advanced software platform for Bayesian evolutionary analysis. PLoS Comput Biol 2019;15:e1006650. 30958812 10.1371/journal.pcbi.1006650 PMC6472827

[btae721-B4] Coles P. Full page screen capture browser extension. https://gofullpage.com/. 2012.

[btae721-B5] Dellicour S , Rose R, Faria NR et al SERAPHIM: studying environmental rasters and phylogenetically informed movements. Bioinformatics 2016;32:3204–6. 27334476 10.1093/bioinformatics/btw384

[btae721-B6] Fick SE , Hijmans RJ. WorldClim 2: new 1-km spatial resolution climate surfaces for global land areas. Int J Climatol 2017;37:4302–15.

[btae721-B7] Hadfield J , Megill C, Bell SM et al Nextstrain: real-time tracking of pathogen evolution. Bioinformatics 2018;34:4121–3. 29790939 10.1093/bioinformatics/bty407 PMC6247931

[btae721-B8] Harris I , Osborn TJ, Jones P et al Version 4 of the CRU TS monthly high-resolution gridded multivariate climate dataset. Sci Data 2020;7:109. 32246091 10.1038/s41597-020-0453-3 PMC7125108

[btae721-B9] He W-T , Bollen N, Xu Y et al Phylogeography reveals association between swine trade and the spread of porcine epidemic diarrhea virus in China and across the world. Mol Biol Evol 2022;39:msab364. 34951645 10.1093/molbev/msab364 PMC8826572

[btae721-B10] Hill SC , Dellicour S, Claro IM et al Climate and land-use shape the spread of zoonotic yellow fever virus. medRxiv. 2022.

[btae721-B11] Ishikawa SA , Zhukova A, Iwasaki W et al A fast likelihood method to reconstruct and visualize ancestral scenarios. Mol Biol Evol 2019;36:2069–85. 31127303 10.1093/molbev/msz131 PMC6735705

[btae721-B12] Kalkauskas A , Perron U, Sun Y et al Sampling bias and model choice in continuous phylogeography: getting lost on a random walk. PLoS Comput Biol 2021;17:e1008561. 33406072 10.1371/journal.pcbi.1008561 PMC7815209

[btae721-B13] Kraemer MUG , Hill V, Ruis C et al; COVID-19 Genomics UK (COG-UK) Consortium. Spatiotemporal invasion dynamics of SARS-CoV-2 lineage B.1.1.7 emergence. Science 2021;373:889–95. 34301854 10.1126/science.abj0113 PMC9269003

[btae721-B14] Layan M , Müller NF, Dellicour S et al Impact and mitigation of sampling bias to determine viral spread: evaluating discrete phylogeography through CTMC modeling and structured coalescent model approximations. Virus Evol 2023;9:vead010. 36860641 10.1093/ve/vead010 PMC9969415

[btae721-B15] Lemey P , Rambaut A, Drummond AJ et al Bayesian phylogeography finds its roots. PLoS Comput Biol 2009;5:e1000520. 19779555 10.1371/journal.pcbi.1000520 PMC2740835

[btae721-B16] Lemey P , Rambaut A, Welch JJ et al Phylogeography takes a relaxed random walk in continuous space and time. Mol Biol Evol 2010;27:1877–85. 20203288 10.1093/molbev/msq067 PMC2915639

[btae721-B17] Nahata KD , Bielejec F, Monetta J et al SPREAD 4: online visualisation of pathogen phylogeographic reconstructions. Virus Evol 2022;8:veac088. 36325034 10.1093/ve/veac088 PMC9615431

[btae721-B18] Sagulenko P , Puller V, Neher RA. TreeTime: maximum-likelihood phylodynamic analysis. Virus Evol 2018;4:vex042. 29340210 10.1093/ve/vex042 PMC5758920

[btae721-B19] Suchard MA , Lemey P, Baele G et al Bayesian phylogenetic and phylodynamic data integration using BEAST 1.10. Virus Evol 2018;4:vey016. 29942656 10.1093/ve/vey016 PMC6007674

