# Supplementary material for: spread.gl: visualizing pathogen dispersal in a high-performance browser application
Source: Bioinformatics. 2024 Nov 29;40(12):btae721. doi: 10.1093/bioinformatics/btae721 (PMC11652268; doi:10.1093/bioinformatics/btae721)
Supplement: btae721_Supplementary_Data [file btae721_supplementary_data.pdf]

Supplementary Figures

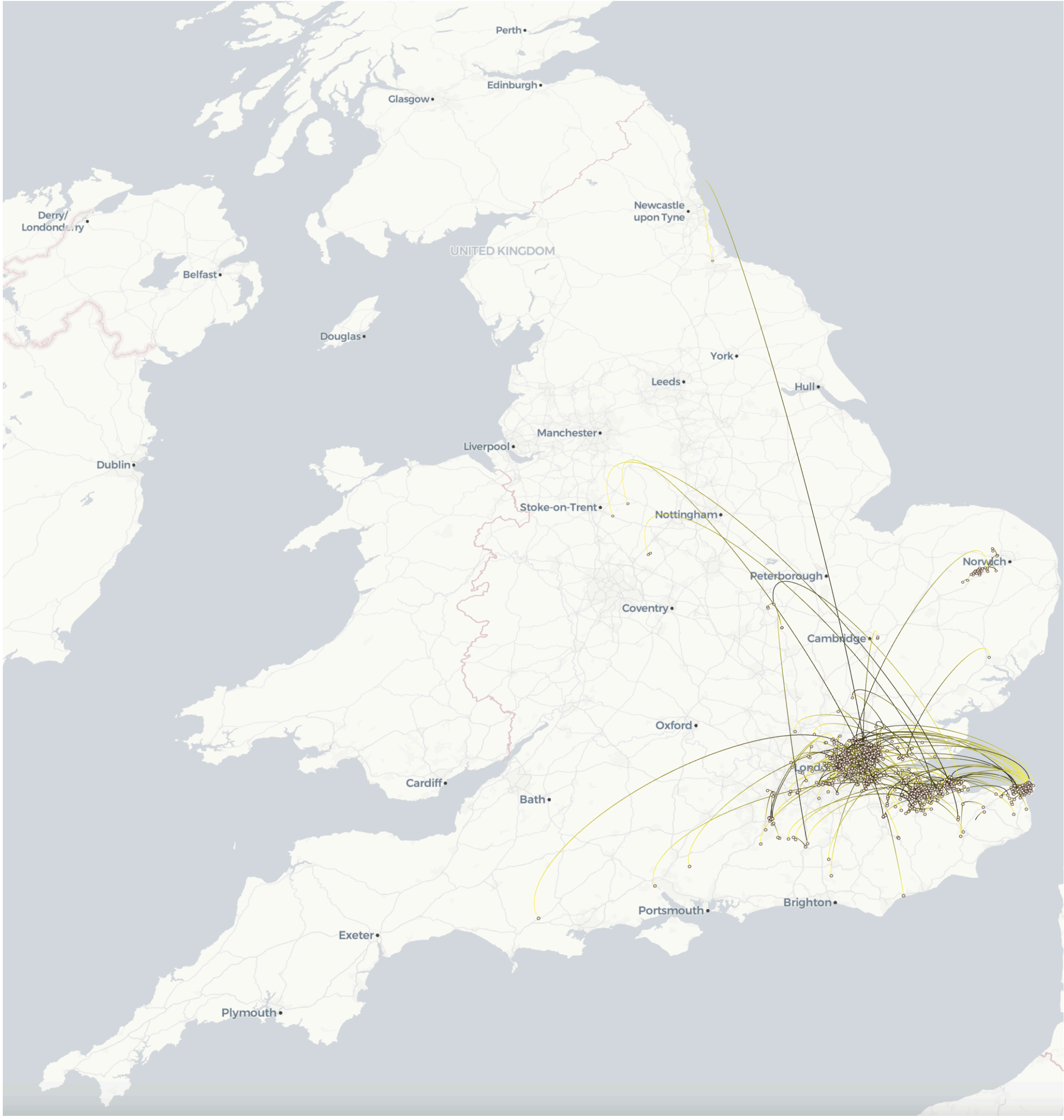

Supplementary Figure S1: Continuous phylogeographic reconstruction of the dispersal history of SARS-CoV-2 lineage B.1.1.7 in England, from September 6th 2020 until November 2nd 2020.

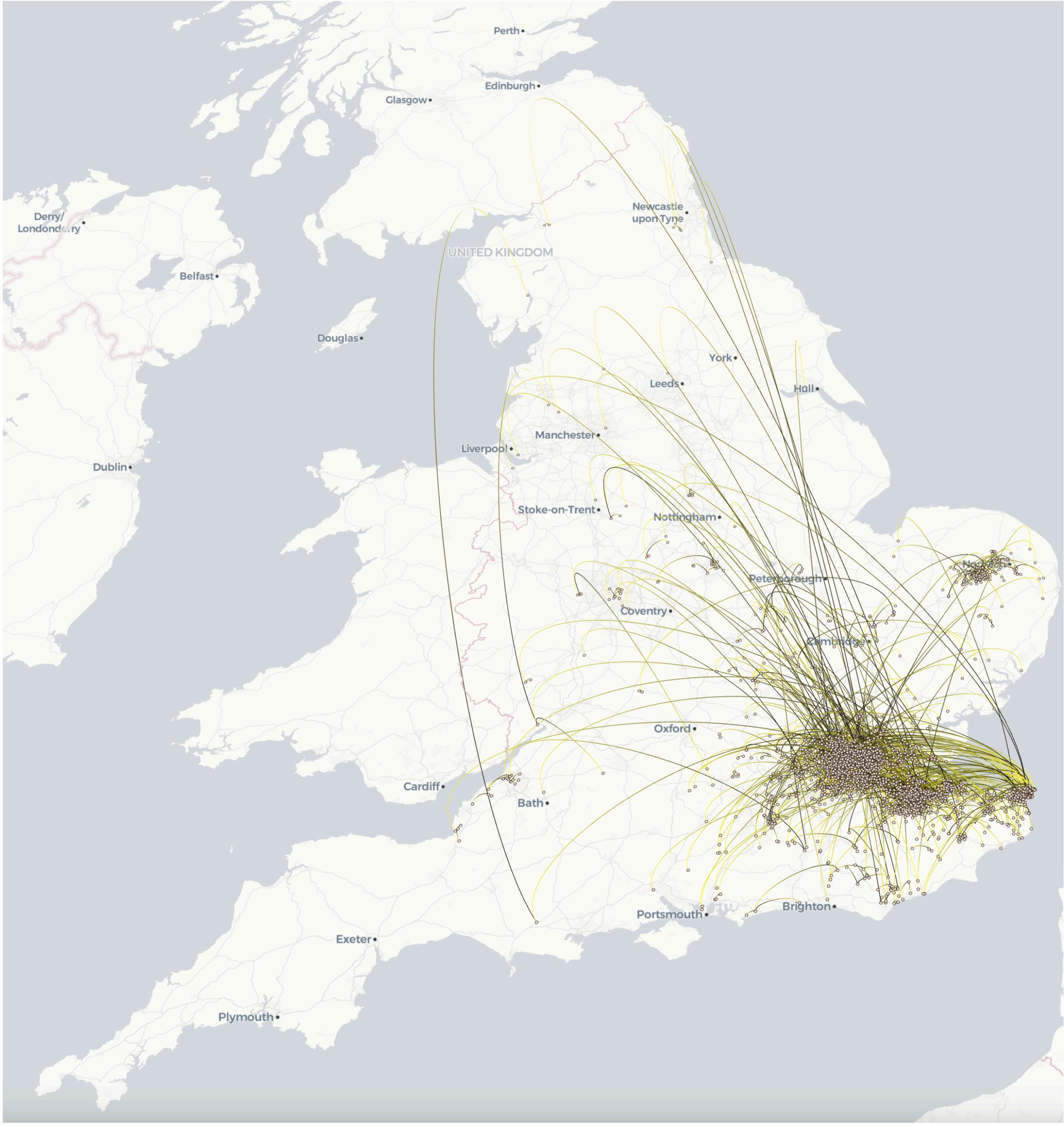

Supplementary Figure S2: Continuous phylogeographic reconstruction of the dispersal history of SARS-CoV-2 lineage B.1.1.7 in England, from September 6th 2020 until November 17th 2020.

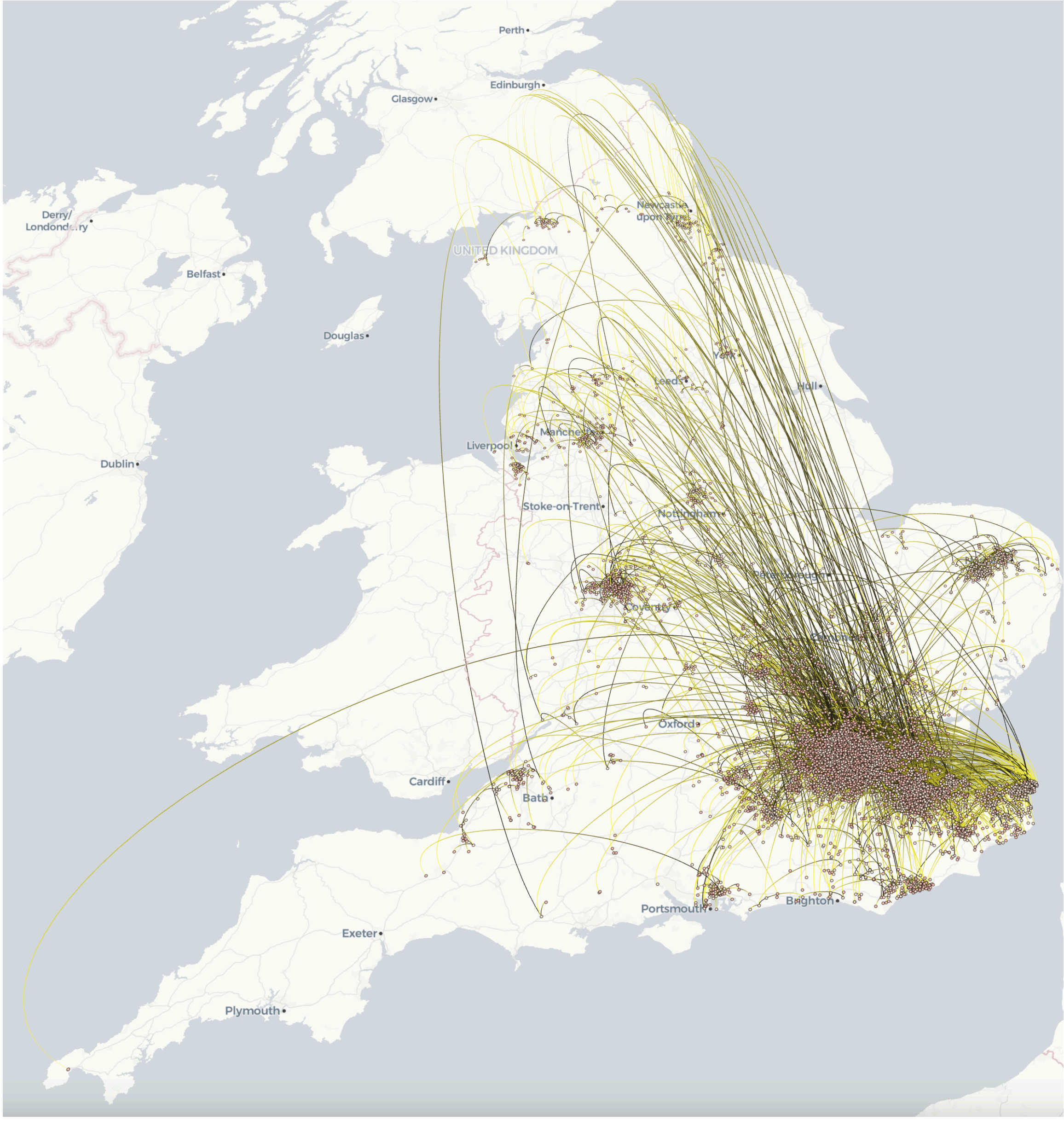

Supplementary Figure S3: Continuous phylogeographic reconstruction of the dispersal history of SARS-CoV-2 lineage B.1.1.7 in England, from September 6th 2020 until December 12th 2020.

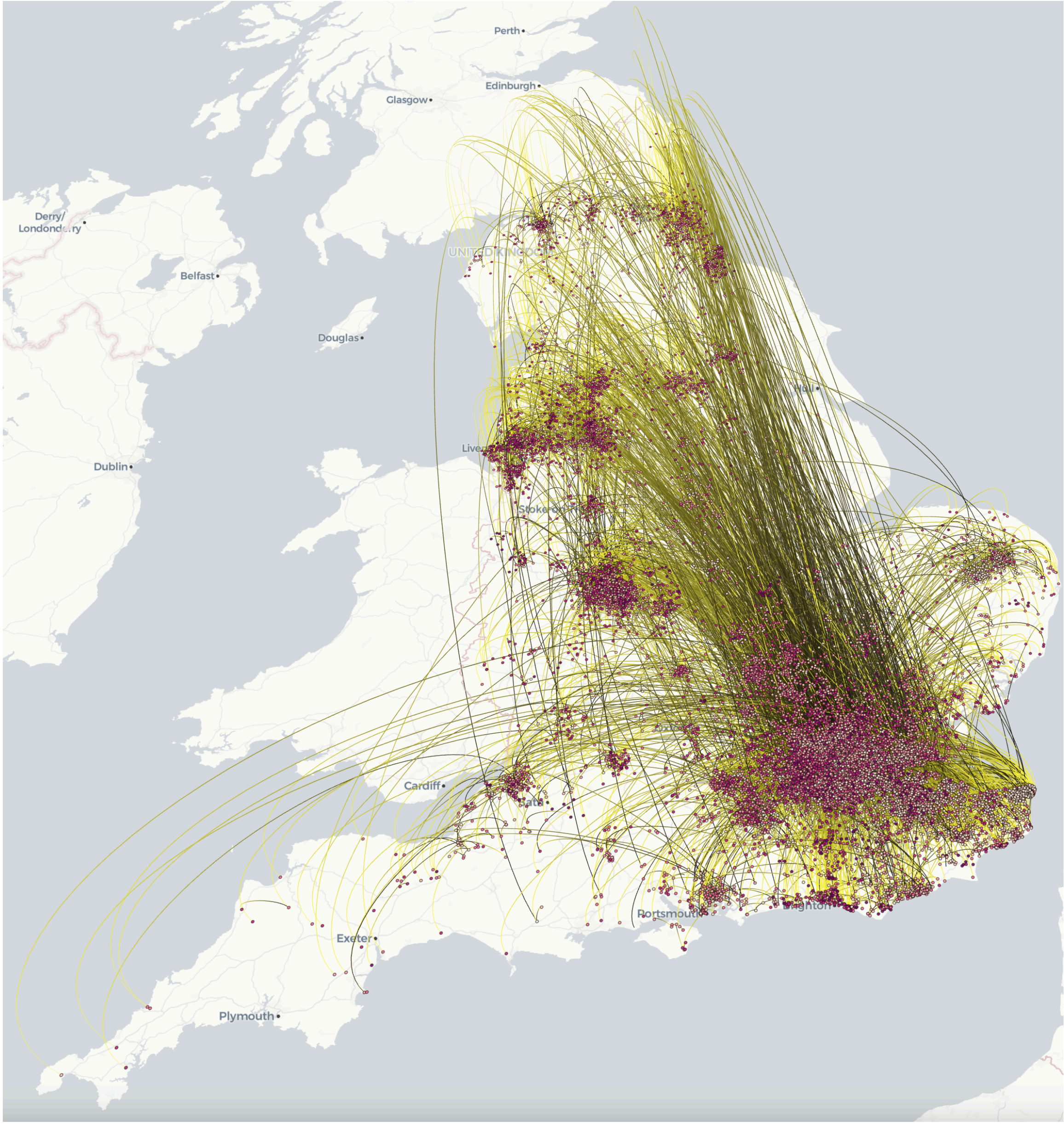

Supplementary Figure S4: Continuous phylogeographic reconstruction of the dispersal history of SARS-CoV-2 lineage B.1.1.7 in England, from September 6th 2020 until January 2nd 2021.
